# Comprehensive preventive treatments for episodic migraine: a systematic review of randomized clinical trials

**DOI:** 10.3389/fneur.2025.1611303

**Published:** 2025-08-18

**Authors:** María-Karina Vélez-Jiménez, Adriana Patricia Martínez-Mayorga, Ildefonso Rodriguez-Leyva, Marisol Jannet Figueroa-Medina, Maria Teresa Reyes-Alvarez, Juan Carlos Pérez-García, Rubén Darío Vargas-García, Daniel San-Juan, Mauricio Pierdant-Perez, Emilio García Gómez, Miguel Angel Morales Morales, Carlos Trenado, Marco Antonio Martínez-Gurrola

**Affiliations:** ^1^Migraine and Headache Clinic, Hospital Angeles Lomas, Mexico City, Mexico; ^2^Department of Neurology, Central Hospital “Dr. Ignacio Morones Prieto”, San Luis Potosi, Mexico; ^3^Faculty of Medicine, Universidad Autónoma de San Luis Potosi, San Luis Potosi, Mexico; ^4^Neurology and Neurosurgery Center, Médica Sur, Mexico City, Mexico; ^5^Christus Murgueza Hospital, Puebla City, Mexico; ^6^Department of Neurology and Psychiatry, Clinica de Mérida, Mérida, Yucatán, Mexico; ^7^Epilepsy Clinic of the National Institute of Neurology and Neurosurgery Manuel Velazco Suárez, Mexico City, Mexico; ^8^Institute of Clinical Neuroscience and Medical Psychology, University Hospital Düsseldorf, Düsseldorf, Germany; ^9^Department of Neurology, Hospital 450, Durango City, Mexico; ^10^Faculty of Medicine, Juarez University of the State of Durango, Durango City, Mexico

**Keywords:** episodic migraine, migraine prevention, CGRP monoclonal antibodies, gepants, non-pharmacological therapy, meta-analysis

## Abstract

**Background:**

Episodic migraine is a prevalent and disabling neurological disorder with a significant impact on quality of life and productivity. Preventive treatment aims to reduce the frequency, intensity, and disability associated with migraine attacks. However, the comparative efficacy and safety of available preventive strategies remain insufficiently addressed in the literature, especially in low- and middle-income countries.

**Objective:**

To evaluate the efficacy and safety of pharmacological and non-pharmacological preventive treatments for episodic migraine through a systematic review and meta-analysis of randomized controlled trials (RCTs).

**Methods:**

Following PRISMA guidelines, a comprehensive literature search was conducted across Wiley Online, BVS, MEDLINE, and OVID databases through November 2024. Eligible studies were RCTs comparing preventive treatments with placebo or active comparators in adults with episodic migraine. This review was not registered in PROSPERO due to institutional constraints at the time of project initiation. Primary outcomes included changes in monthly migraine days (MMD), monthly headache days (MHD), acute medication days (AMD), adverse events (AE) and serious adverse events (SAE). Meta-analysis was performed using fixed- or random-effects models depending on heterogeneity.

**Results:**

Thirty-nine RCTs involving over 15,000 patients were included. Anti-CGRP monoclonal antibodies and gepants demonstrated the most consistent reduction in MMD (−3.2 to −4.4 days) with favorable tolerability. Traditional agents such as topiramate and propranolol showed modest efficacy with higher AE rates. Combination therapies offered superior MMD reductions (up to −5.1 days) but were associated with increased side effects. Non-pharmacological interventions (e.g., neuromodulation, acupuncture) showed promising results but lacked standardization. Meta-analysis of allopathic treatments revealed a significant MMD reduction vs. placebo (−1.25 days; 95% CI − 1.47 to −1.04; *p* < 0.001).

**Conclusion:**

CGRP-targeted therapies and gepants are effective first-line options for episodic migraine prevention. Combinations may enhance efficacy but at the cost of tolerability. Non-pharmacological treatments represent useful adjuncts. These findings support individualized, multimodal preventive strategies, particularly in resource-limited settings. However, interpretation should consider potential publication and language bias, as well as the short follow-up duration in many included trials.

## 1 Introduction

Episodic migraine is a highly prevalent and disabling neurological disorder, particularly among women, affecting millions worldwide (1). It imposes a considerable socioeconomic burden, including lost workdays, decreased productivity, increased healthcare utilization with indirect costs related to caregiving, and diminished quality of life (2, 3). From a clinical standpoint, episodic migraine is characterized by recurrent attacks lasting 4–72 h and occurring on fewer than 15 days per month. These attacks are often unpredictable in onset and severity, resulting in significant physical and emotional distress (2). Effective management requires both acute treatments for symptom relief and preventive strategies aimed at reducing attack frequency and severity over time (4).

Preventive strategies, which range from pharmacological agents like antiseizure medications, beta-blockers, calcitonin gene-related peptide (CGRP) inhibitors, and (anti-CGRP) gepants to non-pharmacological approaches such as cognitive-behavioral therapy and neuromodulation, focus on decreasing the overall burden of the condition (5, 6, 7). Despite significant advances in treatment options, there remains considerable variability in individual responses, highlighting the need for personalized treatment regimens (8).

Although many studies have assessed individual acute and preventive treatments for episodic migraine (4), comprehensive analyses comparing efficacy and safety across multiple pharmacological and non-pharmacological modalities are still lacking, especially in underserved populations (9). Furthermore, global treatment guidelines remain fragmented and inconsistent regarding the integration of emerging therapies, particularly non-pharmacological approaches. This highlights the need for updated evidence-based recommendations applicable across both high- and low-resource healthcare systems. The present study aims to systematically evaluate the efficacy and safety of preventive treatments for episodic migraine in adults, using randomized controlled trials (RCTs) comparing pharmacological or non-pharmacological interventions with placebo or active comparators. A pairwise meta-analysis was performed following PRISMA guidelines, focusing on key outcomes such as monthly migraine days (MMD), monthly headache days (MHD), acute medications days (AMD), and adverse events (AE). Through this work, we aim to inform clinical decision-making and support more equitable guideline development, with a particular focus on relevance for developing countries such as Mexico.

## 2 Materials and methods

This review followed the Preferred Reporting Items for Systematic Reviews and Meta-Analyses (PRISMA) guidelines. This systematic review and meta-analysis was not registered in PROSPERO due to institutional limitations at the time of project initiation. It was part of a broader systematic analysis of preventive treatments for episodic migraine available in Mexico. The study protocol was developed with input from clinical and research experts in headache management. A panel of six neurologists specializing in preventive strategies was assembled to guide protocol development.

Numerous systematic reviews have evaluated the efficacy of various pharmacological treatments, both specific (gepants or monoclonal antibodies) and non-specific (beta-blockers, anti-seizure medications, antidepressants, and others), either individually or in combination, as well as device-based therapies for the prevention of episodic migraine. A systematic review of RCTs was conducted to synthesize the existing evidence on pharmacological and non-pharmacological interventions for episodic migraine.

### 2.1 Data sources and searches

A systematic search was conducted across Wiley Online, BVS, MEDLINE, and OVID from their inception until November 2, 2024. Additional searches included clinical trial registries, government databases and websites, conference proceedings, patient advocacy group websites, systematic reviews/meta-analyses, and medical society websites, though these were ultimately excluded. The technical expert panel assisted in identifying relevant literature. A medical reference librarian designed and executed the search strategy, which was peer-reviewed by a second librarian and validated by coauthors MK, V-J, and I R-L.

### 2.2 Study selection

Eligible studies (1) included adult patients (≥18 years) with episodic migraine; (2) evaluated preventive pharmacologic and non-pharmacological treatments; (3) involved randomized clinical trials (RCTs) (phase II/phase III) comparisons of the intervention with placebo, usual care, another pharmacologic therapy, or no treatment (4) reported outcome of interest as reduction of monthly migraine days (MMD), monthly headache days (MHD) and acute medications days (AMD), (5) adverse events (AE) and serious adverse events (SAE). We excluded *in vitro*, phase I clinical trials, nonrandomized, open-labeled trials, studies without original data, and single-group studies. Therapies in development or intravenous administration terminated development or unavailable in the global market were excluded. Additionally, studies on patients diagnosed with tension headaches or other headache disorders and treated with NSAIDs, triptans, or ergot alkaloids therapies were excluded. Case reports, case series, reviews, post-hoc analyses, or multiple reports of the same study were excluded.

The original study definitions were retained despite evolving migraine criteria, provided they aligned with the current International Classification of Headache Disorders, Third Edition (ICHD-3) standards for episodic migraine (10), characterized by headaches occurring on ≤14 days per month in individuals with migraine. Studies were restricted to those published in English or Spanish.

### 2.3 Data extraction

An extraction form was developed to standardize data collection. Two reviewers independently extracted study characteristics. Inter-rater agreement was assessed using Cohen’s kappa (*κ* = 0.82), indicating substantial agreement. Discrepancies were resolved through consensus discussion. A third reviewer (K.V.) was consulted when necessary. Authors were contacted for clarifications when data were missing or unclear. The extracted data included the generic name of the drug or device, author, year, study design, sample size, intervention details, administration route, dose, frequency, adverse effects, efficacy and safety outcomes, time frame, and availability in Mexico. Treatments were categorized as monotherapy or combination regimens in migraine prevention.

### 2.4 Search strategy

A comprehensive literature search used detailed search terms and Boolean operators to identify relevant studies. The search focused on RCTs that were double-blind AND placebo-controlled interventions. The following pharmacological and non–pharmacological treatment options were included: Erenumab OR CGRP antagonist OR fremanezumab OR galcanezumab OR Eptinezumab OR gepants OR rimegepant OR atogepant OR topiramate OR propranolol OR beta-blocker OR venlafaxine OR valproate OR oxcarbazepine OR candesartan OR amitriptyline OR antiepileptics, OR antidepressants, OR melatonin OR lanepitant OR aspirin OR NSAIDs OR memantine OR neuromodulation OR nerve blockers OR vestibular treatments OR acupuncture. Additionally, the search incorporated studies involving herbal supplements, oils, and combinations using Rayyan© Software, Cambridge, MA, USA.

### 2.5 Outcome measures

The primary efficacy outcome included reducing MMD in the active study group compared with the placebo. The secondary efficacy outcomes were MHD and reduction of AMD, which include specific and non-specific substances. When data on reduction in days or standard deviations were not directly reported in the articles, they were estimated based on comparisons between baseline and final values, reported percentage changes, or visual inspection of figures. Standard deviations were calculated from reported standard errors or visually estimated when necessary. The primary safety outcome included the presence and frequency or percentage of adverse effects and SAE; type, and severity of adverse effects using the Common Terminology Criteria for grading from Grade 1 (mild) to Grade 5 (death), and availability in Mexico.

### 2.6 Risk of bias assessment

The risk of bias was evaluated using the Cochrane Risk of Bias Tool for Randomized Trials (RoB 2, v2) (11). This assessment covered five key domains: (1) bias in randomization procedures, (2) bias from deviations in intended interventions, (3) bias due to incomplete outcome data, (4) bias in outcome measurement, and (5) bias in selective reporting. Each domain was rated as “low risk,” “some concerns,” or “high risk,” with an overall bias judgment assigned per study. For this analysis, MMD were the primary outcome to determine bias. Two independent reviewers (D.S. and M.A.M.M.) conducted the assessments.

### 2.7 Statistical analysis

Data analysis was performed with SPSS software (v.31; IBM Corp., Armonk, NY, USA). Heterogeneity across studies was evaluated using the Chi-square test and quantified via the *I*^2^ statistic. A fixed-effect model was applied for analyses with low heterogeneity (*I*^2^ < 50%), while a random-effects model was used when *I*^2^ ≥ 50%. No subgroup or sensitivity analyses were pre-specified due to the limited number of homogeneous studies available. Funnel plots were generated to assess publication bias for the primary efficacy comparison (allopathic pharmacological treatments vs. placebo for MMD). Egger’s test was applied and showed no evidence of publication bias (*p* = 0.27). Continuous outcomes were expressed as mean differences (MD) with 95% confidence intervals (CIs). Statistical significance was defined as *p* < 0.05. The meta-analyses conducted in this review were limited to efficacy outcomes, specifically MMD, due to heterogeneity in the reporting and classification of AE and SAE across studies.

## 3 Results

### 3.1 Characteristics of the included studies

**Figure 1:**
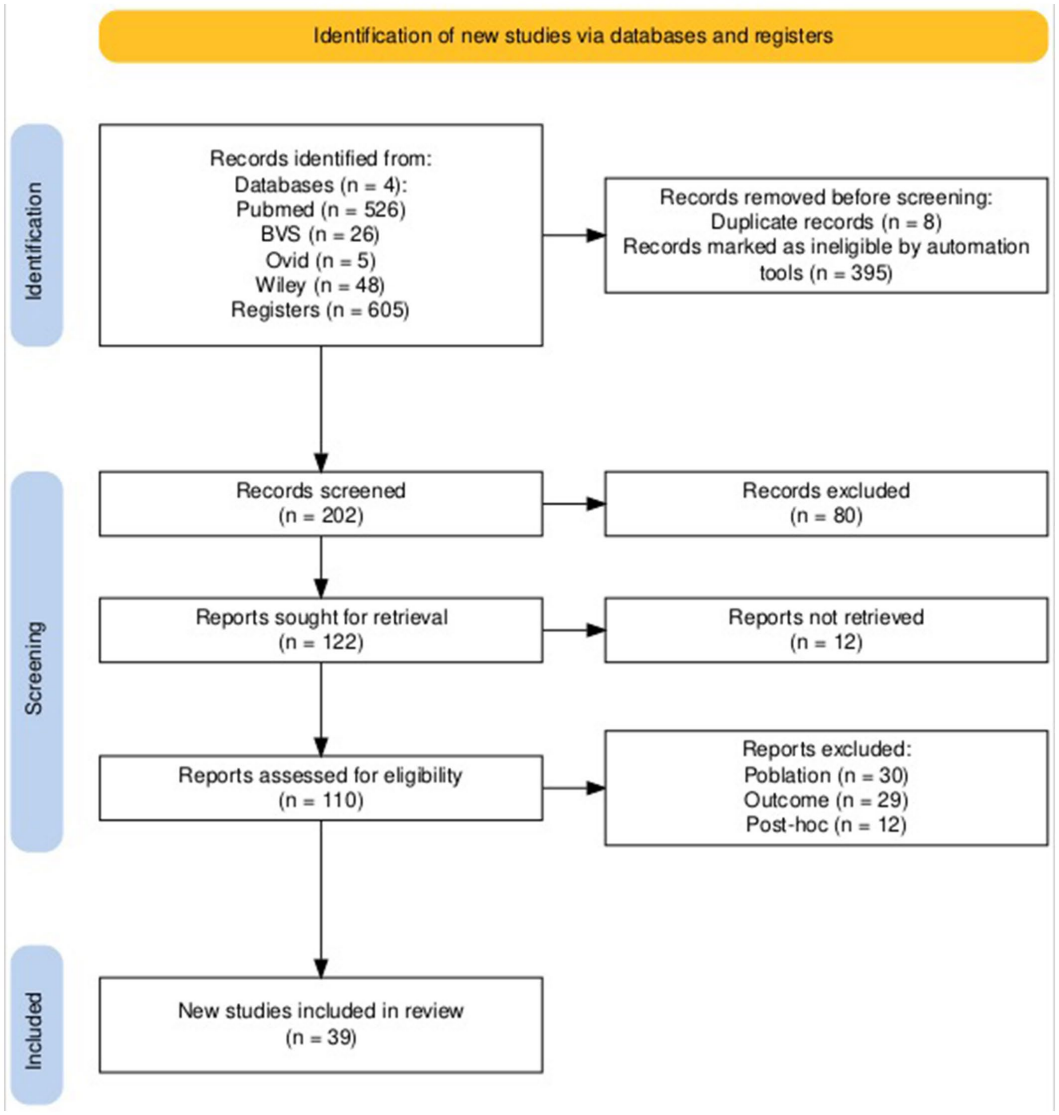
Flowchart of the randomized clinical trials analyzed.

Our search until November 30, 2024, identified 605 scientific papers through database and trial registry screening; after removing duplicates or illegible by automation tools, 202 records remained. Figure 1 shows the flowchart of the clinical studies screened and excluded, and finally, 39 RCTs were included and analyzed. All studies were published between 1987 and 2024. All studies were randomized double-blinded clinical trials classified in pharmacological treatments and non-pharmacological interventions.

### 3.2 Study designs and frequencies

The included studies predominantly employed RCT designs, with the majority (27) utilizing double-blind, placebo-controlled methodologies. Additionally, 12 studies implemented double-blind RCTs with active comparators, directly comparing the efficacy and safety of different pharmacological treatments.

### 3.3 Sample size distribution

The included studies demonstrated considerable variability in sample sizes ranging from 28 to 1,001 participants, with a distinct trend toward larger trials (>300 participants) evaluating newer therapeutic classes such as anti-CGRP monoclonal antibodies (e.g., erenumab, fremanezumab) and gepants (e.g., rimegepant, atogepant). In contrast, smaller-scale trials were more frequently observed for established drug classes, including anti-seizure medications (e.g., topiramate, valproate) and beta-blockers (e.g., propranolol), as well as non-pharmacologic approaches such as acupuncture.

### 3.4 Study settings

All interventions were administered in outpatient or clinical trial environments, with routes including oral, subcutaneous (SC), intravenous (IV), transcutaneous, and topical.

### 3.5 Efficacy and safety of pharmacologic therapies

Table 1 summarizes the study design, sample size, interventions, clinical outcomes, adverse events profile, and availability in Mexico. This section presents a structured narrative synthesis of the efficacy and safety findings of each pharmacologic class used for episodic migraine prevention.

**Table 1:** Summary of the efficacy and safety of randomized clinical trials in preventing episodic migraine.

Episodic migraine preventive treatment									
Generic drug name, formulation	Author, year, and ref*	Study design and sample size	Main intervention (route, dose & frequency)	Outcome					
				MMD (monthly migraine days)	MHD (monthly headache days)	AMD (acute medication days)	AE (adverse events)	SAEs (serious adverse events)	Available in Mexico (yes/no)
CGRP antagonist									
Erenumab, SC	Goadsby, P. J. et al. (2017) (12)	RCT double-blind, placebo-controlled, 955 participants 317 erenumab 70 mg 319 erenumab 140 mg 319 placebo	Erenumab, SC 70 mg 140 mg Placebo SC	At week 12 −70 mg: −3.2 (SD ± 3.5) −140 mg: −3.7 (SD ± 3.5) Placebo: −1.8 (SD ± 3.5)	No reported	At week 12 −70 mg: −1.1 (SD ± 1.7) −140 mg: −1.6 (SD ± 1.7) Placebo: −0.2 (SD ± 1.7)	Erenumab: −70 mg: 57% −140 mg: 55% -upper respiratory tract infection -nasopharyngitis -sinusitis Placebo: 63% -upper respiratory tract infection -nasopharyngitis -sinusitis	Erenumab: −70 mg: 2.5% −140 mg: 1.9% -upper respiratory tract infection -nasopharyngitis -sinusitis Placebo: 2.2% -upper respiratory tract infection -nasopharyngitis -sinusitis	Yes
Fremanezumab, SC	Dodick et al., 2018 (13)	RCT double-blind, placebo-controlled, 875 participants 290 fremanezumab monthly 291 fremanezumab single dose 294 placebo	SC 225 mg monthly 675 mg single dose	At week 12 -Monthly: −3.7 (SD ± 4.0) -Single dose: −3.4 (SD ± 4.0) Placebo: −2.2 (SD ± 4.0)	No reported	At week 12 -Monthly: −3.0 (SD ± 2.5) -Single dose: −2.9 (SD ± 2.5) Placebo: −1.6 (SD ± 2.5)	Fremanezumab: -Monthly: 47% -Single dose: 44% Placebo: 40% -Injections site reactions	Fremanezumab: 1.3% -Depression -Anxiety Placebo: 2% -Injections site erythema -Injection site induration	Yes
Galcanezumab, SC	Skljarevski et al., 2018 (14)	RCT double-blind, placebo-controlled, 915 participants	SC 120 mg 240 mg monthly	At 6 months −120 mg: −4.3 (SD ± 4.5) −240 mg: −4.2 (SD ± 4.4) placebo: −2.3 (SD ± 4.2)	No reported	At 6 months −120 mg: −3.7 (SD ± 3.0) −240 mg: −3.6 (SD ± 2.9) placebo: −1.9 (SD ± 4.2)	Galcanezumab: 58% -Injections site reactions Placebo: 56% -Injections site reactions	Galcanezumab: −120 mg: 2.2% −240 mg: 4.0% -Injection site reactions -pruritic rash -bronchiectasis Placebo: 1.7% -Injection site reactions	Yes
Eptinezumab, IV	Smith et al., 2020 (15)	RCT double-blind, placebo-controlled, 888 participants 223 eptinezumab 30 mg 221 eptinezumab 100 mg 222 eptinezumab 300 mg 222 placebo	IV 30 mg 100 mg 300 mg every 12 weeks	At week 12 Eptinezumab 30 mg: −4.0 (SD ± 8.0) Eptinezumab 100 mg: −3.9 (SD ± 8.0) Eptinezumab 300 mg: −4.3 (SD ± 8.0) Placebo: −3.2 (SD ± 8.0)	No reported	No reported	Eptinezumab 100 mg: 21.2% Eptinezumab 300 mg: 18.5% -upper respiratory tract infections -sinusitis -fatigue -Nausea Placebo: 17.3% -upper respiratory tract infections -sinusitis -fatigue -Nausea	No reported	Yes
Gepants									
Rimegepant, oral	Croop et al., 2021 (16)	RCT double-blind, placebo-controlled, 747 participants 373 rimegepant 374 placebo	Oral 75 mg once daily	At week 12 Rimegepant: −4.3 (SD ± 4.6) Placebo: −3.5 (SD ± 4.6)	No reported	At week 12 Rimegepant: −3.7 (SD ± 5.2) Placebo: −4.0 (SD ± 5.2)	Rimegepant: 35.6% -Nausea -fatigue -upper respiratory tract infections Placebo: 34.9% -Nausea -fatigue -upper respiratory tract infections	Rimegepant: 1.2% -AMI -CVD -allergic reaction Placebo: 1.5% -AMI -CVD -allergic reaction	Yes
Atogepant, oral	Goadsby et al., 2020 (17)	RCT double blind placebo controlled 825 participants 93 atogepant 10 mg 183 atogepant 30 mg 186 atogepant 60 mg 86 atogepant 30 mg twice 91 atogepant 60 mg twice 186 placebo	Oral 10 mg 30 mg 60 mg once daily	At week 12 Atogepant: 10 mg: −4.0 (SD ± 2.8) 30 mg: −3.8 (SD ± 2.7) 60 mg: −3.6 (SD ± 2.6) 30 mg (twice): −4.2 (SD ± 3.5) 60 mg (twice): −4.1 (SD ± 3.5) Placebo: −2.9 (SD ± 2.6)	At week 12 Atogepant: 10 mg: −4.3 (SD ± 3.8) 30 mg: −4.2 (SD ± 4.0) 60 mg: −3.9 (SD ± 3.9) 30 mg (twice): −4.2 (SD ± 3.5) 60 mg (twice): −4.3 (SD ± 3.5) Placebo: −2.9 (SD ± 4.00)	At week 12 Atogepant: 10 mg: −3.7 (SD ± 2.8) 30 mg: −3.9 (SD ± 2.7) 60 mg: −3.5 (SD ± 2.6) 30 mg (twice): −3.8 (SD ± 2.6) 60 mg (twice): −3.6 (SD ± 2.6) Placebo: −2.4 (SD ± 2.6)	Atogepant: 26% -nausea, -fatigue - Constipation Placebo: 16% -nausea, -fatigue - Constipation	No reported	Yes
Atogepant, oral	Ailani et al., 2021 (18)	RCT double-blind, placebo-controlled, 873 participants 214 atogepant 10 mg 223 atogepant 30 mg 222 atogepant 60 mg 214 placebo	Oral 10 mg 30 mg 60 mg once daily	At week 12 Atogepant: 10 mg: −3.7 (SD ± 2.9) 30 mg: −3.9 (SD ± 2.9) 60 mg: −4.2 (SD ± 2.9) Placebo: −2.5 (SD ± 2.9)	At week 12 Atogepant: 10 mg: −3.9 (SD ± 2.9) 30 mg: −4.0 (SD ± 2.9) 60 mg: −4.2 (SD ± 2.9) Placebo: −2.5 (SD ± 2.9)	At week 12 Atogepant: 10 mg: −3.7 (SD ± 2.9) 30 mg: −3.7 (SD ± 2.9) 60 mg: −3.9 (SD ± 2.9) Placebo: −2.4 (SD ± 2.9)	Atogepant: 10 mg: 63.8% 30 mg: 61.5% 60 mg: 62.3% -Nausea -Constipation Placebo: 54.6% -Nausea -Constipation	No reported	Yes
Atogepant, oral	Tassorelli et al., 2024 (19)	RCT double-blind, placebo-controlled, 309 participants 154 atogepant 155 placebo	Oral 60 mg once daily	At week 12 Atogepant: −4.2 (SD ± 4.9) Placebo: −1.9 (SD ± 4.9)	No reported	No reported	Atogepant: 10% -Constipation -Fatigue -Nausea Placebo: 3% -Constipation -Fatigue -Nausea	Atogepant: 2% -Constipation Placebo: 1% -Nausea	Yes
Atogepant, oral	Schwedt et al., 2022 (20)	RCT double-blind, placebo-controlled, 873 participants 214 atogepant 10 mg 223 atogepant 30 mg 222 atogepant 60 mg 214 placebo	Oral 10 mg 30 mg 60 mg once daily	At week 12 Atogepant: 10 mg: −4.2 (SD ± 2.9) 30 mg: −4.3 (SD ± 3.0) 60 mg: −4.4 (SD ± 2.9) Placebo: −3.0 (SD ± 2.9)	At week 12 Atogepant: 10 mg: −4.2 (SD ± 2.9) 30 mg: −4.2 (SD ± 3.0) 60 mg: −4.4 (SD ± 3.0) Placebo: −3.0 (SD ± 1.9)	At week 12 Atogepant: 10 mg: −3.3 (SD ± 2.9) 30 mg: −3.4 (SD ± 3.0) 60 mg: −3.7 (SD ± 3.0) Placebo: −1.7 (SD ± 2.9)	Atogepant: 53.7% -Constipation -Fatigue -Nausea Placebo: 56.8% -Constipation -Fatigue -Nausea	Atogepant: 4.1% -Nausea -Fatigue Placebo: 2.7% -Fatigue	Yes
Anti-seizure medications									
Topiramate vs. propranolol, oral	Ashtari, Shaygannejad and Akbari, 2008 (21)	RCT double-blind, 60 participants 30 Topiramate 30 Propranolol	Oral topiramate: 25–50 mg daily propranolol: 40–80 mg daily	At week 8 Topiramate: −4.2 (SD ± 1.2) Propranolol: −3.6 (SD ± 0.9)	No reported	No reported	Topiramate: 60% -Paresthesia, -weight loss -somnolence -dizziness Propranolol: 50% -bradycardia, −hypotension -dizziness.	No reported	Yes
Valproate extended-release (Divalproex), oral vs. placebo	Freitag et al., 2002 (22)	RCT double-blind, placebo-controlled, 237 participants	Oral Valproate extended release 500–1,000 mg daily	At week 4 Valproate extended release: −1.7 (SD ± 0.4) Placebo: −0.7 (SD ± 0.4)	At week 4 Valproate extended release: −1.2 (SD ± 0.2) Placebo: −0.6 (SD ± 0.2)	No reported	Valproate extended release: 68% -Infection -Nausea -Asthenia -Flu -Dyspepsia -Diarrhea Placebo: 45% -Nausea -Diarrhea -Flu	Valproate extended release: 2% -Nausea Placebo: 1% -Nausea	Yes
Oxcarbazepine, oral	Silberstein et al., 2008 (23)	RCT double-blind, placebo-controlled, 170 participants 85 oxcarbazepine 85 placebo	Oral Oxcarbazepine 300–1,200 mg daily	At week 12 Oxcarbazepine: −1.3 (SD ± 2.6) Placebo: −1.7 days (SD ± 2.6)	No reported	At week 12 Oxcarbazepine: −1.2 (SD ± 3.7) Placebo: −2.1 (SD ± 3.7)	Oxcarbazepine: 80% -Fatigue -Dizziness - Nause. Placebo: 65% -Fatigue -Dizziness -Somnolence	Oxcarbazepine: 10.6% -Acute vestibulopathy Placebo: 4.7% days -Depression	Yes
Topiramate, oral	Storey et al., 2001 (24)	RCT double-blind, placebo-controlled, 40 participants 19 Topiramate 21 Placebo	Oral 25–200 mg daily	At week 20 Topiramate: −1.8 (SD ± 2.2) Placebo: −0.5 (SD ± 2.8)	No reported	No reported	Topiramate: 72% -paresthesia -weight loss -memory impairment -emotional lability -abnormal vision Placebo: 40% -Drowsiness -Nausea -Gastrointestinal intolerance	Topiramate: 15% -Nausea -Emotional lability Placebo: No reported	Yes
Beta-blockers									
Propranolol long-acting, oral	Pradalier et al., 1989 (25)	RCT double-blind, placebo-controlled, 74 participants 40 Propranolol 34 Placebo	Oral 160 mg daily	At week 12 160 mg: −2.9 (SD ± 1.2) Placebo: −0.4 (SD ± 2.1)	No reported	No reported	Propranolol: 80 mg: 50% 160 mg: 60% -Tiredness -Dizziness placebo: 40% -Tiredness -Dizziness	No reported	Yes
Propranolol vs. N-alpha methyl histamine, oral	Millán-Guerrero et al., 2014 (26)	RCT is controlled with another active arm, 60 participants 30 Propranolol 30 placebo	Oral propranolol: 80 mg n-alpha methyl histamine: 10 mg	At week 12 N-alpha Methyl Histamine: −2.0 (SD ± 0.2) Propranolol: −4.2 (SD ± 0.1)	No reported	No reported	N-alpha Methyl Histamine: 45% -Reactions in the injection site Propranolol: 55% -Reactions in the injection site	No reported	Yes
Metoprolol vs. placebo	Steiner et al., 1987 (27)	RCT double-blind placebo-controlled 59 participants 28 Metoprolol 31 placebo	Oral Metoprolol 50–100 mg BID	At week 4 Metoprolol: −1.2 (SD ± 0.6) Placebo: −0.4 (SD ± 0.2)	No reported	No reported	Metoprolol: 17.9% -Nightmares -Weight increase -Disenea Placebo: 12.9% -Drowsiness -Vertigo -Pruritis	Metoprolol: 3.5% -Heartburn Placebo: No reported	Yes
Metoprolol vs. placebo	Kangasniemi et al., 1987 (28)	RCT double-blind placebo-controlled cross-over	Oral Metoprolol slow-release 200 mg daily	At week 8 Metoprolol: −2.0 Placebo: −1.3	No reported	No reported	Metoprolol: 36% -fatigue -gastrointestinal disturbances -sleep disturbances Placebo: 18% -fatigue -gastrointestinal disturbances	No reported	Yes
Metoprolol vs. propranolol	Olsson et al., 2009 (29)	RCT double-blind cross-over study 56 participants	Oral Metoprolol 50 mg BID Propranolol 40 mg BID daily	At week 8 Metoprolol: −1.2 (SD ± 1,4) Placebo: −1.2 (SD ± 1.4)	No reported	No reported	Metoprolol: 36% -fatigue -gastrointestinal disturbances -sleep disturbances Propranolol: 18% -fatigue -gastrointestinal disturbances -sleep disturbances	No reported	Yes
Metoprolol vs. nevibolol	Schellenberg et al., 2007 (30)	RCT double-blind 30 participants 14 metoprolol 16 nebivolol	Oral Metoprolol 47.5–95 mg Nevibolol 5 mg daily	At week 14 Metoprolol: −2.1 (SD ± 1.4) Nevibolol: −1.7 (SD ± 2.1)	No reported	No reported	Metoprolol: 93% -fatigue -bradycardia Nevibolol: 69% -fatigue -bradicardia	Metoprolol: 7.1% -migraine deterioration Nevibolol:6.2% -sleep disturbances	Yes
Angiotensin receptor blocker									
Candesartan vs. propranolol slow release vs. placebo	Stovner et al., 2013 (31)	RCT triple-blind placebo-controlled, double cross-over study 72 participants 59 candesartan 61 propranolol 61 placebo	Oral Candesartan: 60 mg Propranolol: 160 mg daily	At week 12 Candesartan: −1.8 (SD ± 4.1) Propranolol slow release: −1.9 (SD ± 4.2) Placebo: −1.2 (SD ± 4.2)	At week 12 Candesartan: −2.9 (SD ± 4.1) Propranolol slow release: −1.9 (SD ± 4.2) Placebo: −2.2 (SD ± 4.2)	No reported	Candesartan: 50% -Respiratory tract infections -Dizziness -Bodily pain -Sleep problems Propranolol slow release: 58% -Respiratory tract infections -Bodily pain -Dizziness -Sleep problems Placebo: 33% -Respiratory tract infections -Bodily pain -Sleep problems -Diarrhoea	No reported	Yes
Antidepressants									
Amitriptyline vs. valproate, oral	Kalita, Bhoi and Misra, 2013 (32)	RCT double-blind 300 participants 150 amitriptyline 150 valproate	Oral amitriptyline: 20 mg twice a day valproate: 500 mg daily	At week 12 amitriptyline: −7.8 (SD ± 0.5) Divalproate: −6.9 (SD ± 2.7)	No reported	No reported	Amitriptyline: 22% -Drowsiness -Dry mouth Divalproate: 18% -Weight gain -Nausea	Amitriptyline: 1% -Severe sedation Divalproate: 3% -Liver enzyme elevation	Yes
Amitriptyline ER, oral	Lampl et al., 2009 (33)	RCT double-blind 132 participants 66 low dose 66 high dose	Oral amitriptyline: 25 mg daily amitriptyline: 50 mg daily	At week 12 Amitriptyline low dose: −1.0 (SD ± 2.0) Amitriptyline high dose: −1.0 (SD ± 2.0)	No reported	No reported	Amitriptyline low dose: 12% -Mild sedation Amitriptyline high dose: 18% -Mild sedation -Dry mouth -Weight gain	Amitriptyline low doses: No reported Amitriptyline high dose: 1% -Sedation	No
Venlafaxine vs. placebo	Ozyalcin et al., 2005 (34)	RCT double-blind placebo-controlled 60 participants 21 venlafaxine 150 mg 20 venlafaxine 75 mg 19 placebo	Oral venlafaxine 75 mg venlafaxine 150 mg daily	At week 8 venlafaxine 75 mg: −1.8 (SD ± 1.3) venlafaxine 150 mg: −2.0 (SD ± 1.5) placebo: −0.9 (SD ± 1.9)	No reported	No reported	venlafaxine 75 mg:100% -Nausea -somnolence -fatigue venlafaxine 150 mg: 95.2% -Nausea -somnolence -fatigue placebo: 55.6% -Nausea -fatigue -dizziness	No reported	Yes
Venlafaxine vs. escitalopram	Tarlaci et al., 2009 (35)	RCT double-blind 93 participants 35 venlafaxine 58 escitalopram	Oral venlafaxine 72.8 mg escitalopram 12.4 mg daily	At week 12 venlafaxine: −3.8 (SD not available) escitalopram: −2.6 (SD not available)	No reported	No reported	Venlafaxine: 28.6% -Nausea -somnolence -dizziness Escitalopram: 0%	Venlafaxine: 34.3% -Nausea -somnolence -dizziness	Yes
Venlafaxine vs. amitriptyline	Bulut et al., 2004 (36)	RCT double-blind cross-over 52 participants 26 venlafaxine 26 amitriptyline	Oral venlafaxine amitriptyline	At week 12 venlafaxine: −2.3 (SD ± 1.8) amitriptyline: −1.7 (SD ± 1.9)	No reported	No reported	Venlafaxine: 23% -Nausea -tachycardia -others Amitriptyline: 80% -hypersomnia -dry mouth	Venlafaxine: 3.2% -nausea -taquicardia -others Amitriptyline: 19.2% -hypersomnia -orthostatic hypotension	Yes
Hormonal therapy									
Melatonin, oral	Alstadhaug et al., 2010 (37)	RCT double-blind cross over placebo-controlled, 48 participants	Oral 2 mg daily	At week 8 Melatonin: −2.8 (SD ± 1.6) Placebo: −2.9 (SD ± 1.4)	No reported	No reported	Melatonin: 2.8% -Fatigue -Dizziness -Nervousness Placebo: 4.7% -Eczema -Fatigue -Dry mouth	No reported	Yes
NK-1 receptor antagonist									
Lanepitant, oral	Goldstein et al., 2001 (38)	RCT double-blind placebo-controlled 84 participants 42 lanepitant 42 placebo	Oral 200 mg daily	At week 12 Lanepitant: −0.9 (SD ± 3.7) Placebo: −0.5 (SD ± 3.4)	No reported	No reported	Lanepitant: 52.4% -Headache -Back pain -Diarrhea Placebo: 40.5% -Headache -Back pain -Diarrhea	Lanepitan: 2.3% -Nausea -Heart plapitations Placebo: 4.7% -Insomnia -Confusion	No
NSAIDs									
Acetylsalicylic acid, oral	Benseñor et al., 2001 (39)	RCT double-blind, placebo-controlled, 1,001 participants	Oral 81 mg daily	At 36 months Aspirin: −1.3 (SD ± 2.0) Placebo: −1.5 (SD ± 1.9)	No reported	No reported	No Reported	No reported	Yes
NMDA antagonist									
Memantine, oral	Noruzzadeh et al., 2016 (40)	Double-blind RCT 52 participants 25 memantine 27 placebo	Oral 10 mg daily	At week 12 Memantine −3.5 (SD ± 1.6) Placebo: −0.8 (SD ± 2.1)	No reported	No reported	Memantine: 13% -Dizziness -Fatigue -Nausea Placebo: 3.7% -Nausea	Memantine: No reported Placebo: 3.7% -Vertigo	Yes
Neuromodulator									
Occipital nerve stimulation vs. sham	Liu et al., 2017 (41)	RCT 110 participants 22 tONS 2 Hz 22 tONS 100 Hz 22 tONS 2/100 Hz 22 sham 22 Topiramato	Transcutaneous frequencies: 2 Hz 100 Hz 2/100 Hz daily	No reported	At 1 month: 2 Hz: −2.0 (SD ± 1.4) 100 Hz: −5.5 (SD ± 1.4) 2/100 Hz: −3.0 (SD ± 1.4) Sham: −0.5 (SD ± 1.4) TPM: −6.0 (SD ± 1.4)	No reported	tONS 2 Hz: 25% -Pain and hematoma tONS 100 Hz: 20% -Pain and hematoma tONS 2/100 Hz: 22% -Pain and hematoma -Sham: 18% -Pain and hematoma	No reported	No
Caloric vestibular stimulation	Wilkinson et al., 2017 (42)	RCT 81 participants	30 min each daily	At week 12 CVS: −3.9 (SD ± 2.7) Placebo: −1.1 (SD ± 3.9)	No reported	At week 12 CVS: −3.9 (SD ± 3.2) Placebo: −1.7 (SD ± 6.1)	CVS: -Nausea -Dizziness -Ear discomfort -Tinnitus Placebo: -Nausea -Dizziness -Ear discomfort -Tinnitus	No reported	Yes
Acupuncture	Alecrim-Andrade et al., 2006 (43)	RCT 28 participants 14 real acupuncture 14 sham acupuncture	Acupuncture sessions twice weekly	At week 12 Acupuncture: −2.5 (SD ± 2.6) Sham acupuncture: −1.0 (SD ± 3.3)	No reported	No reported	Acupuncture: 25% -Pain -Hematoma Sham acupuncture: 20% -Pain	No reported	Yes
Herbal supplements and oil									
*Tanacetum parthenium*, oral	Pfaffenrath et al., 2002 (44)	Double-blind, RCT 147 participants 37 Tanacetum 2.08 mg 36 Tanacetum 6.25 mg 39 tanacetum 18.75 mg 35 placebo	Oral 2.08 mg 6.25 mg 18.75 mg three times daily	At week 12 *Tanacetum parthenium* 2.08 mg: −0.2 (SD ± 1.2) 6.25 mg: −0.9 (SD ± 1.7) 18.75 mg: −0.4 (SD ± 1.7) Placebo: −0.7 (SD ± 1.8)	No reported	No reported	*Tanacetum parthenium*: 35% -Nausea -Diarrhea Placebo: 35% -Nausea -Diarrhea	No reported	No
Ginger, oral	Martins et al., 2020 (45)	Double-blind RCT 107 participants 53 Ginger 54 placebo	Oral 200 mg three times daily	At week 12 Ginger: −0.9 (SD ± 2.1) Placebo: −0.7 (SD ± 2.2)	At week 12 Ginger: −08 (SD ± 2.9) Placebo: −0.5 (SD ± 2.9)	At week 12 Ginger: −0.9 (SD ± 2.1) Placebo: −0.6 (SD ± 1.4)	Ginger: 30% Placebo: 14.8% -Heartburn -Nausea -Constipation	Ginger: 7.5% Placebo: 1.8% -Heartburn -Nausea -Constipation	No
Basil Essential Oil, topic	Ahmadifard et al., 2020 (46)	Triple-blind, RCT 144 participants 36 basil oil 2% 36 basil oil 4% 36 basil oil 6% 36 placebo	Topic 2, 4, 6% 3 times daily	At week 12 Basil essential oil 2%: −2.8 (SD ± 1.8) 4%: −3.0 (SD ± 1.8) 6%: −3.2 (SD ± 1.8) Placebo: −1.0 (SD ± 1.8)	No reported	No reported	Oil: 8.3% -Skin irritation Placebo: 2.7%	No reported	No
Combinations									
Topiramate and amitriptyline (alone or in combination), oral	Keskinbora and Aydinli, 2008 (47)	Double-blind, RCT 63 participants 20 Topiramate 22 Amitriptyline 21 Combination	Oral topiramate: 50–200 mg daily vs. amitriptyline: 10–150 mg daily	At week 12 Topiramate: −5.6 (SD ± 3.3) Amitriptyline: −5.1 (SD ± 2.7) Combination: −5.1 (SD ± 2.8)	No reported	No reported	Topiramate: 30% -Paresthesia -Fatigue -Loss of appetite Amitriptyline: 25% -Sedation -Dry mouth Combination: 35% -Dizziness -Weight gain -Fatigue	Topiramate: 10% -Paresthesia -Loss of appetite Amitriptyline: 8% -Drowsiness Combination: 4.3% -Sedation -Dizziness	Yes
Topiramate and flunarizine (alone or in combination), oral	Luo et al., 2012 (48)	Double-blind, RCT 126 participants 39 Flunarizine 44 Topiramate 43 Combination	Oral topiramate: 50–10 mg daily flunarizine: 5–10 mg daily	At week 12 Topiramate: −3.4 (SD ± 1.6) Flunarizine: −3.1 (SD ± 1.5) Combination: −2.6 (SD ± 0.8)	No reported	No reported	Topiramate: 25% -Memory disturbances -Paresthesia -Fatigue -Weight loss Flunarizine: 20.5% -Drowsiness -Weight gain -Gastrointestinal disturbances Combination: 23.3% -Sedation -Fatigue	No reported	Yes
Topiramate plus nortriptyline, oral	Krymchantowski, Da Cunha Jevoux, and Bigal, 2012 (49)	RCT double-blind, placebo-controlled, 80 participants 17 Topiramate 19 Nortriptyline 44 Combination	Oral topiramate: 50 mg 100 mg daily nortriptyline: 25–75 mg daily	At week 6 Topiramate: −3.5 (SD ± 2.3) Nortriptyline: −3.2 (SD ± 2.3) Combination: −4.6 (SD ± 1.9)	No reported	No reported	Combination: 65.9% -Weight loss -Dry mouth -Paresthesia -Somnolence Placebo: 41.2% -Weight loss -Weight gain	No reported	Yes
Propranolol and nortriptyline (alone or in combination), oral	Domingues et al., 2009 (50)	Double-blind RCT 44 participants 14 Propranolol 14 Nortriptyline 16 Combination	Oral propranolol: 40 mg daily nortriptyline: 25 mg daily	At week 8 Propranolol: −4.0 (SD ± 3.9) Nortriptyline: −1.0 (SD ± 4.3) Combination: −4.0 (SD ± 4.1)	No reported	No reported	Propranolol: 18% -Fatigue Nortriptyline: 22% -Drowsiness -Dry mouth Combination: 15% -Dizziness -Mild sedation	Propranolol: 5% -Fatigue Nortriptyline: 6% -Drowsiness Combination: 3% -Dizziness -Sedation	Yes

#### 3.5.1 Anti-CGRP monoclonal antibodies

Anti-CGRP monoclonal antibodies (erenumab, fremanezumab, galcanezumab, eptinezumab) showed consistent reductions in MMD, ranging from −3.2 to −4.3 days, mostly evaluated at 12 weeks, except for galcanezumab which was assessed at 6 months. AMD was reduced between −1.1 and −3.7 days within the same timeframe. MHD was not consistently reported. AE rates ranged from 18.5 to 58%, with upper respiratory infections, injection site reactions, constipation, and fatigue being most common. SAEs occurred in 1.3–4%, including abdominal pain, asthenia, and bronchiectasis.

#### 3.5.2 Gepants

Gepants (rimegepant and atogepant) demonstrated significant reductions in MMD (−3.6 to −4.4 days), MHD (−3.9 to −4.4 days), and AMD (−3.3 to −3.9 days), all consistently evaluated at 12 weeks. AE ranged widely (10–63.8%), mainly nausea, constipation, and fatigue. SAE occurred in 2–4.1%, including allergic reactions and fatigue.

#### 3.5.3 Antiseizure medications

Topiramate, valproate, and oxcarbazepine showed heterogeneous evaluation periods: topiramate was assessed at 8–20 weeks, valproate at 4 weeks, and oxcarbazepine at 12 weeks. MMD reductions ranged from −1.3 to −4.2 days. Valproate showed a −1.2-day reduction in MHD at 4 weeks, and oxcarbazepine showed a −1.2-day reduction in AMD at 12 weeks. AE were frequent (60–80%), particularly paresthesia, weight loss, dizziness, and cognitive effects. SAE ranged from 2 to 15%.

#### 3.5.4 Beta-blockers

Propranolol, metoprolol, and nebivolol showed MMD reductions from −1.2 to −4.2 days over evaluation periods ranging from 4 to 14 weeks. MHD and AMD were not reported. AE frequency varied significantly (17.9–93%), with fatigue, dizziness, and gastrointestinal issues being most common. SAE ranged from 3.5 to 7.1%.

#### 3.5.5 Angiotensin receptor blockers

Candesartan showed a reduction in MMD (−1.8 days) and MHD (−2.9 days), both measured at 12 weeks. AMD data was not reported. AE frequency reached 50%, mainly respiratory infections, dizziness, and sleep problems. No SAE were specified.

#### 3.5.6 Antidepressants

Amitriptyline, venlafaxine, and escitalopram were evaluated over 8–12 weeks. MMD reductions ranged from −1.0 to −7.8 days. MHD and AMD were not reported. AE incidence ranged widely (0–100%), often including drowsiness, fatigue, nausea, and weight gain. SAE ranged from 1 to 34.3%, with severe sedation and cardiovascular symptoms in some cases.

#### 3.5.7 Hormonal therapy

Melatonin was evaluated over 8 weeks, showing an MMD reduction of −2.8 days. MHD and AMD were not reported. AE occurred in 2.8% of cases, including fatigue and dizziness. No SAE were reported.

#### 3.5.8 NK-1 receptor antagonists

Lanepitant was evaluated over 12 weeks and showed a modest MMD reduction of −0.9 days. No data was available for MHD or AMD. AE were reported in 52.4% of participants, including headache and gastrointestinal symptoms. SAE occurred in 2.3% of participants.

#### 3.5.9 NSAIDs

Aspirin (acetylsalicylic acid) showed a −1.3-day reduction in MMD after long-term evaluation at 36 months. MHD, AMD, AE, and SAE data were not reported.

#### 3.5.10 NMDA antagonists

Memantine was evaluated over 12 weeks, achieving an MMD reduction of −3.5 days. AMD remained unchanged (0 days), and MHD was not reported. AE (13%) included dizziness and fatigue. SAE were not specified.

#### 3.5.11 Herbal supplements and oils

Tanacetum, ginger, and basil oil were studied over 12 weeks. MMD reductions ranged from −0.2 to −3.2 days. Ginger also led to a reduction in MHD (−0.8 days) and AMD (−0.9 days). AE frequencies varied (8.3–35%) and included nausea, diarrhea, and skin irritation. Ginger was associated with a 7.5% SAE rate.

#### 3.5.12 Combination therapies

Combinations such as topiramate with amitriptyline, flunarizine, or nortriptyline were evaluated between 6 and 12 weeks. MMD reductions ranged from −2.6 to −5.1 days. MHD and AMD data were mostly unavailable. AE frequency ranged from 15 to 65.9%, and SAE reached 4.3%, including sedation, weight gain, and dizziness.

Among the evaluated pharmacological groups, Gepants (Rimegepant, Atogepant) demonstrated one of the most substantial reductions in both Monthly Migraine Days (MMD) and Acute Medication Days (AMD), with MMD decreasing between −3.6 to −4.4 days and AMD showing a reduction of −3.3 to −3.9 days at 12 weeks. Similarly, anti-CGRP monoclonal antibodies (Erenumab, Fremanezumab, Galcanezumab, Eptinezumab) exhibited notable efficacy, achieving MMD reductions ranging from −3.2 to −4.3 days and AMD reductions of −1.1 to −3.7 days across various studies. Additionally, combination therapies such as Topiramate + Amitriptyline or Propranolol + Nortriptyline presented the most significant MMD reduction, with values reaching up to −5.1 days, though AMD data was not reported for this group. These findings highlight the potential of these medication classes in effectively reducing migraine frequency and medication use.

### 3.6 Meta-analysis of efficacy of the pharmacological treatments

#### 3.6.1 Allopathic medications

**Figure 2:**
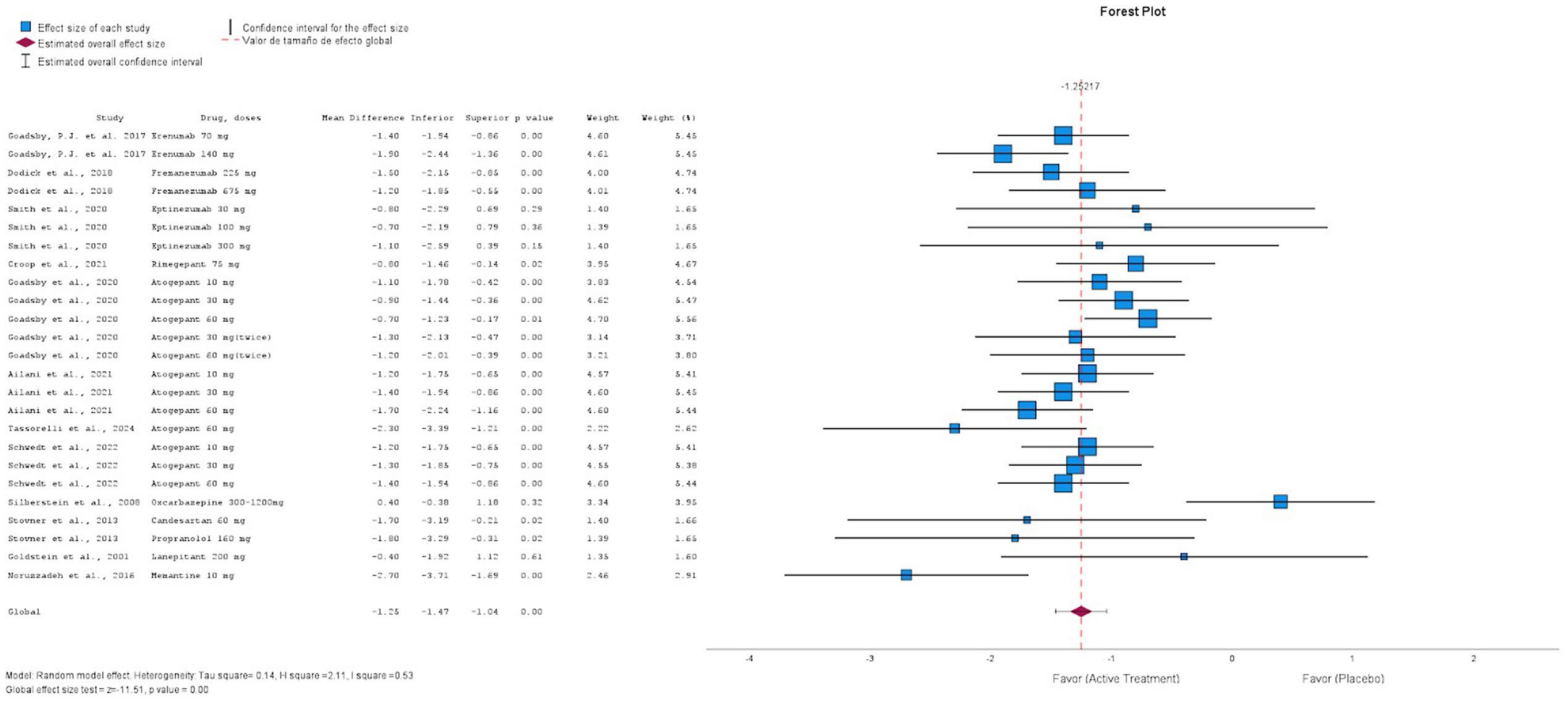
Forest plot of the randomized clinical trials placebo-controlled using allopathic treatments for the prevention of episodic migraine.

**Figure 3:**
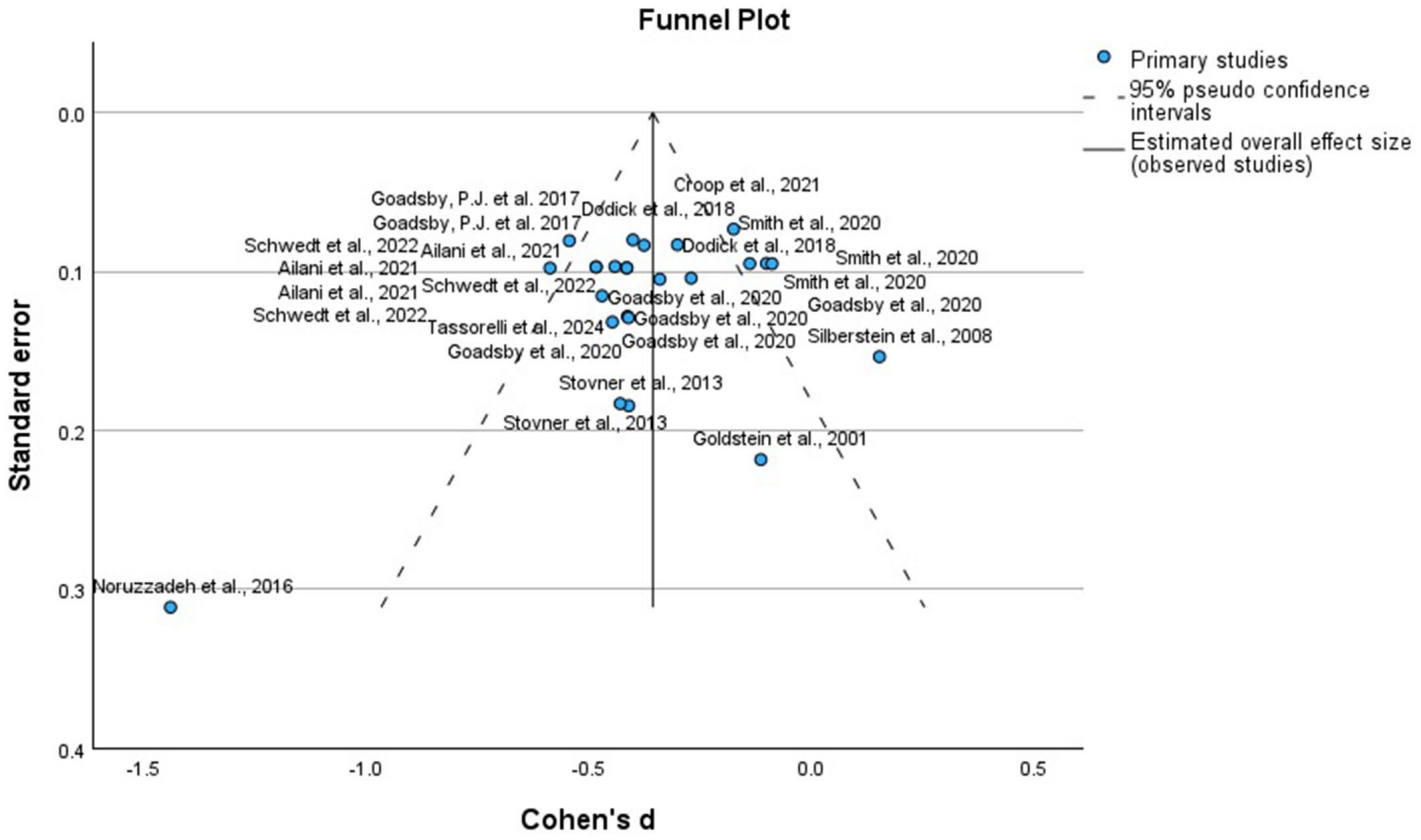
Funnel plot of the randomized clinical trials, placebo-controlled using allopathic treatments to prevent episodic migraine.

We analyzed the RCTs that compared the intervention with placebo at the 12 weeks, using MMD as the primary outcome, as it is the most consistent measure of efficacy. To reduce heterogeneity in the timing of outcome evaluations, we excluded RCTs with different times for the assessment of the outcomes, active and heterogeneous comparators, and study arms that involved drug combinations previously mentioned in Table 1. Figure 2 of allopathic treatment showed a global −1.25 mean difference (95%, confidence interval CI −1.47, −1.04, *p* = 0.001) to favor the active treatments, except in some negative RCTs using eptinezumab, oxcarbazepine, candesartan, propranolol, and lanepitant. Although the pooled mean reduction in monthly migraine days (MMD) was −1.25 days, this effect should be interpreted in light of the baseline MMD observed in the placebo groups, which typically ranged from 4.5 to 7.5 days across the included trials. This corresponds to a relative reduction of approximately 17–28%, indicating a potentially meaningful clinical benefit despite the modest absolute value. Figure 3 displays the funnel plot corresponding to this meta-analysis of allopathic treatments versus placebo for MMD. Visual inspection showed no significant asymmetry, and Egger’s test did not detect publication bias. It is important to note that the quantitative meta-analysis focused exclusively on efficacy outcomes (MMD), and pooled estimates for AE or SAE were not calculated due to significant variability in reporting methods and definitions among included studies.

#### 3.6.2 Homeopathic medications

**Figure 4:**
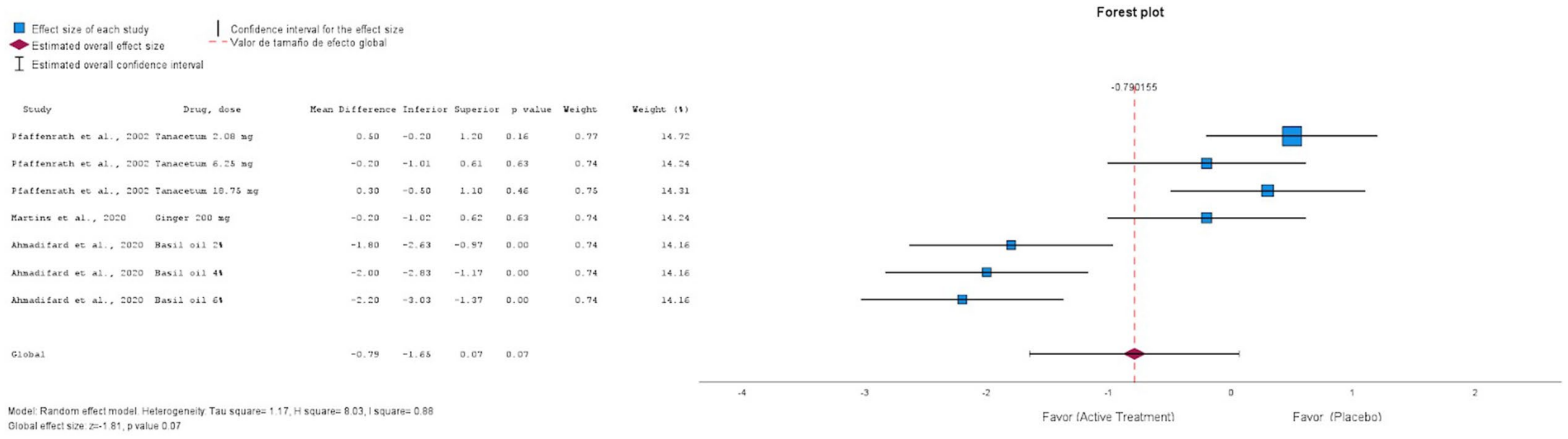
Forest plot of the randomized clinical trials placebo-controlled using homeopathic treatments for the prevention of episodic migraine.

**Figure 5:**
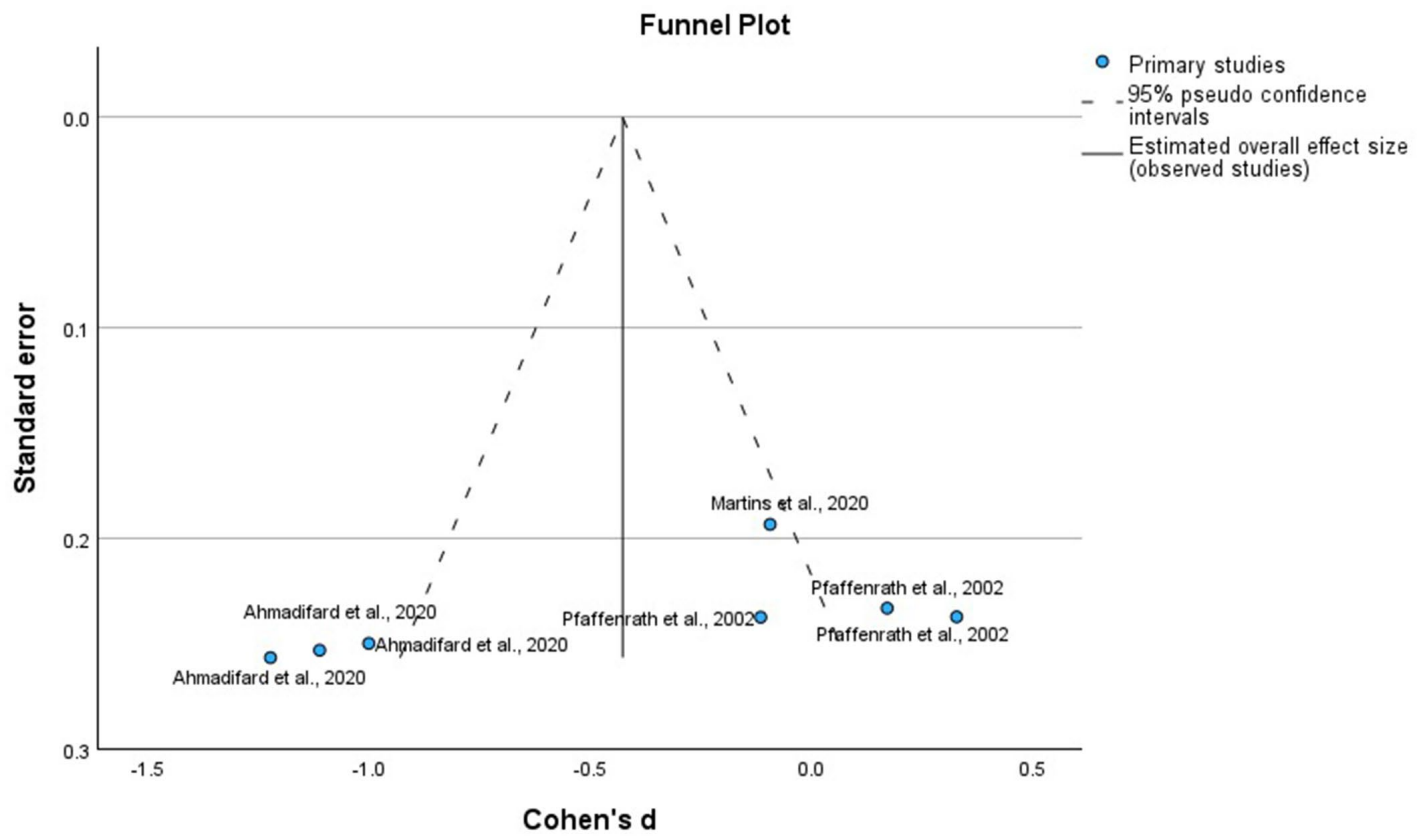
Funnel plot of the randomized clinical trials placebo-controlled using homeopathic treatments for the prevention of episodic migraine.

We analyzed the RCTs that compared the intervention with placebo at 12 weeks, using MMD as the primary outcome. Figure 4 shows that homeopathic treatments had a global mean difference of −0.79 (95% confidence interval [CI]: −1.65 to 0.07, *p* = 0.07), which was not significant compared with the placebo. The funnel plot, shown in Figure 5 corresponds to the meta-analysis of homeopathic treatments. Visual inspection showed no major asymmetry, and Egger’s test did not indicate significant publication bias.

### 3.7 Efficacy and safety of non-pharmacologic therapies

Table 2 summarizes a structured narrative synthesis of non-pharmacological. This section provides a structured narrative synthesis of non-pharmacological interventions for episodic migraine prevention.

**Table 2:** Summary of non-pharmacological therapies.

Intervention	Evaluation period	MMD (days)	MHD (days)	AMD (days)	AE	SAE
Occipital Nerve Stimulation (ONS)	1 month	Not reported	−2.0 to −5.5	Not reported	Pain, hematoma, nausea, dizziness (up to 25%)	Not reported
Caloric Vestibular Stimulation (CVS)	12 weeks	−3.9	−3.9	−3.9	Nausea, dizziness, ear discomfort, tinnitus	Not reported
Acupuncture	12 weeks	−2.5	Not reported	Not reported	Pain and hematoma at puncture sites (25%)	Not reported

Non-pharmacological interventions included neuromodulation techniques such as occipital nerve stimulation, caloric vestibular stimulation, and acupuncture. These modalities were assessed primarily over a 12-week period, except for occipital nerve stimulation, which reported outcomes at 1 month.

Occipital nerve stimulation showed reductions in MHD ranging from −2.0 to −5.5 days at 1 month, depending on the stimulation frequency. MMD also improved, although the data were not consistently reported across all frequency subgroups. AE were reported in up to 25% of patients and included local pain, hematoma at the stimulation site, nausea, and dizziness. SAE were not reported in these trials.

Caloric vestibular stimulation was evaluated over 12 weeks and demonstrated a reduction in both MMD and MHD of −3.9 days. The same intervention showed a reduction in AMD of −3.9 days. The most frequent AE included nausea, dizziness, ear discomfort, and tinnitus. No SAE were reported.

Acupuncture, evaluated over a 12-week period, showed a reduction of −2.5 days in MMD compared to a −1.0-day reduction in the sham acupuncture control group. MHD and AMD were not reported. AE occurred in 25% of patients and included local pain and hematoma at the puncture sites. No SAE were reported.

Although these interventions yielded promising effects in reducing migraine frequency and associated medication use, the wide variability in protocols, outcome definitions, and follow-up times hindered direct comparison and aggregation of results. Nonetheless, the generally favorable safety profile across studies supports the potential role of these non-pharmacological strategies as adjunctive treatments in individualized preventive regimens.

Unfortunately, the meta-analysis of the efficacy of the non-pharmacological treatments was not feasible for the heterogeneity of the outcome measurements used in each study.

### 3.8 GRADE evidence profile

A structured GRADE assessment was conducted to determine the certainty of evidence for the main pharmacological comparisons included in this review. This approach complements the narrative synthesis and meta-analysis by addressing potential limitations in risk of bias, inconsistency, indirectness, imprecision, and publication bias.

Table 3 summarizes the GRADE evidence profiles for anti-CGRP monoclonal antibodies, gepants, and combination therapies, using monthly migraine days (MMD) as the primary outcome.

**Table 3:** GRADE evidence profiles.

Comparison	Outcome	Number of studies	Risk of bias	Inconsistency	Indirectness	Imprecision	Publication bias	Certainty	Effect estimate	Reason for downgrading
Anti-CGRP monoclonal antibodies vs placebo	MMD at 12 weeks	4 RCTs	Not serious	Not serious	Not serious	Serious (some wide CIs)	Unlikely	Moderate	−3.2 to −4.3 days	Imprecision due to variability in sample size and confidence intervals
Gepants vs placebo	MMD at 12 weeks	5 RCTs	Not serious	Not serious	Not serious	Not serious	Unlikely	High	−3.6 to −4.4 days	None
Combination therapy vs monotherapy	MMD	3 RCTs	Some concerns	Serious (high heterogeneity)	Serious (limited generalizability)	Serious (small sample size)	Possible	Low	−2.6 to −5.1 days	Small sample sizes and variation in comparator drugs

The certainty of evidence was rated as moderate for anti-CGRP therapies due to some imprecision across trials, high for gepants based on robust and consistent findings, and low for combination treatments, mainly due to heterogeneity, indirect comparisons, and small sample sizes. These ratings provide a useful framework for interpreting the strength and applicability of the observed clinical effects.

### 3.9 Risk of bias

**Figure 6:**
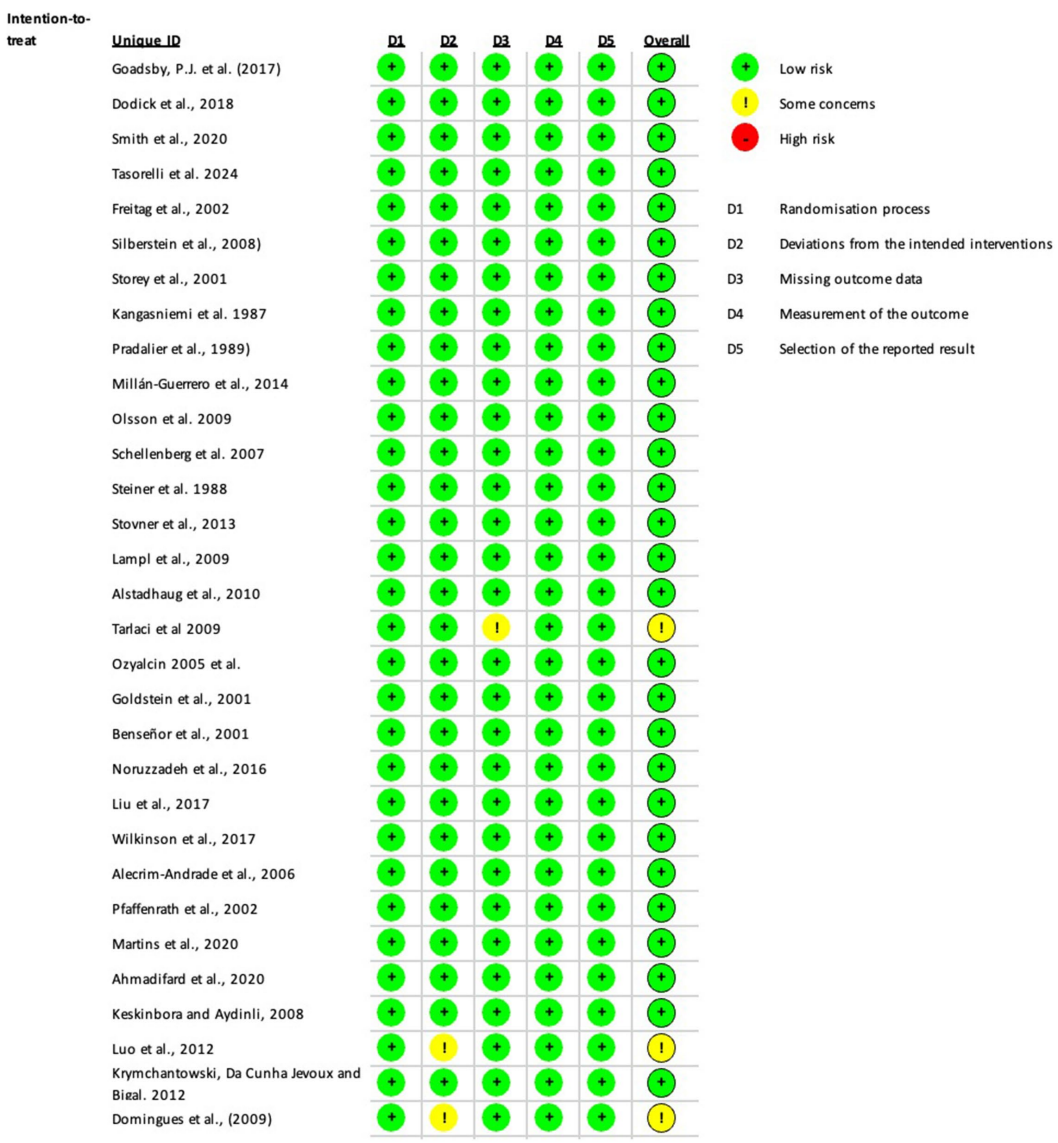
Risk of bias for the randomized clinical trials included intention-to-treat.

**Figure 7:**
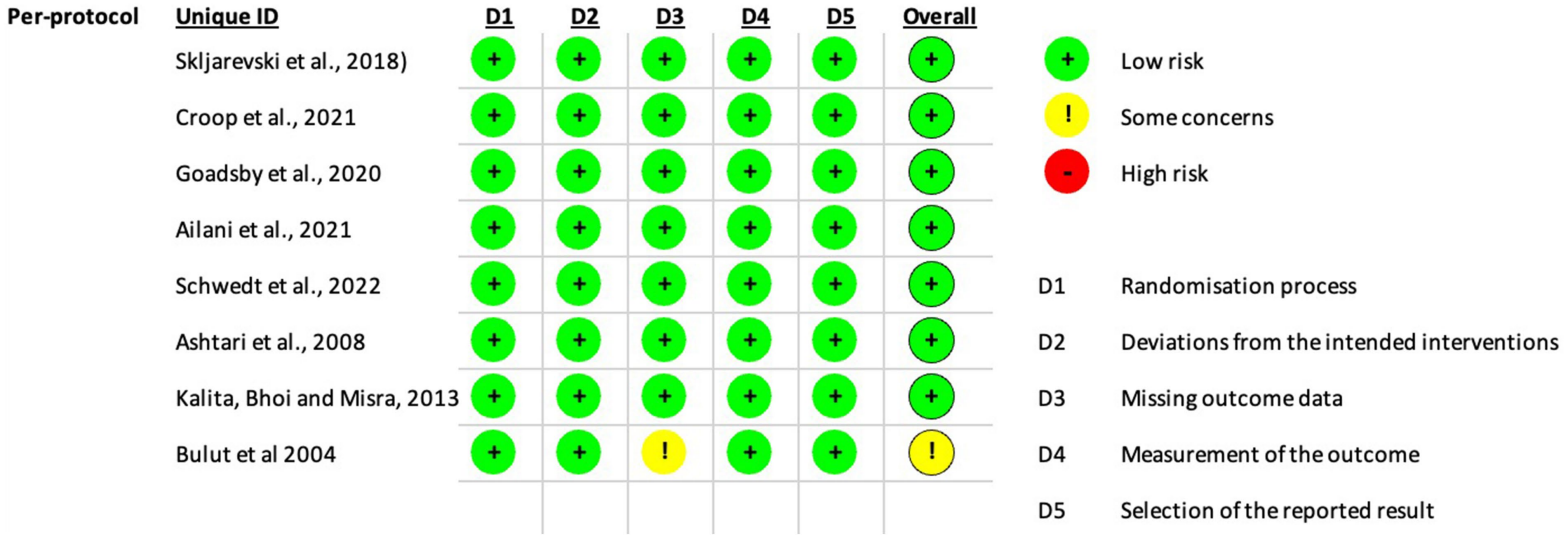
Risk of bias for the randomized clinical trials included per protocol.

Bias was evaluated following the guidelines of the Cochrane Handbook for Systematic Reviews. The details are illustrated in Figures 6, 7. We did not find any risk of bias in the RCTs using the intention-to-treat modality. However, in the Per-protocol approach, participants were randomly assigned to groups using computer-generated random sequences through an interactive web-response system in all two studies. One trial noted that pharmacists were unblinded; however, their role was limited to drug preparation and inventory management. Another trial did not provide details on allocation concealment and blinding of outcome assessment. All two trials reported patient follow-up losses; each predefined outcome was clearly described. Studies meeting the inclusion criteria were included in this meta-analysis.

## 4 Discussion

This systematic meta-analysis provides a comprehensive evaluation of the efficacy and safety of preventive interventions for episodic migraine, with a particular focus on those available in Mexico. Although the allopathic treatment analysis demonstrated an overall favorable effect for active interventions (mean difference: –1.25; 95% CI: −1.47 to −1.04; *p* = 0.001), several agents—including eptinezumab, oxcarbazepine, candesartan, propranolol, and lanepitant—showed non-significant effects in individual RCTs, highlighting variability in therapeutic response. The findings confirm that, despite the wide range of therapeutic options, there remains significant heterogeneity in clinical outcomes regarding reduction in MMD and treatment tolerability.

Anti-CGRP antagonists—including erenumab, fremanezumab, galcanezumab, and eptinezumab—demonstrated clinically relevant reductions in MMD with acceptable safety profiles (51). These results are consistent with international literature, where anti-CGRP monoclonal antibodies have shown superiority over traditional treatments regarding specificity and treatment adherence.

Similarly, gepants (rimegepant and atogepant), as oral CGRP receptor modulators, showed comparable efficacy to monoclonal antibodies, albeit with variability in adverse events, particularly gastrointestinal side effects (52).

Regarding conventional therapies, antiepileptic drugs and beta-blockers, despite their widespread use, showed more modest efficacy and higher rates of adverse events, limiting their applicability to specific patient profiles (53). Notably, pharmacological combinations—such as topiramate with amitriptyline or propranolol with nortriptyline—achieved the most pronounced reductions in MMD, although with lower tolerability, underscoring the need for individualized risk–benefit assessment (54, 55).

Non-pharmacological interventions, including occipital nerve stimulation, vestibular stimulation, and acupuncture, demonstrated beneficial effects on some clinical outcomes. However, the lack of uniformity in outcome measures and the limited number of controlled studies hindered their inclusion in the quantitative meta-analysis. Nevertheless, their favorable safety profile and potential utility as adjunctive therapies warrant further exploration through studies with robust methodological design (56).

In light of the limited availability of certain pharmacological agents and therapies in Mexico, it is imperative to outline strategic steps for incorporating newer evidence-based treatments into national formularies, ensuring equitable access and alignment with international standards of care.

From a methodological perspective, the risk of bias analysis using the Cochrane RoB 2 tool revealed an overall low risk of bias, particularly in studies that used intention-to-treat analysis (57). However, some limitations persisted, especially in studies with limited information on allocation concealment or blinding of outcome assessors.

The limitation of the present analysis is the exclusion of patients over 65 years of age, pregnant women, and individuals with cardiovascular or cerebrovascular conditions, which restricts the generalizability of the findings. Furthermore, variability in follow-up periods (ranging from 3 to 6 months), potential publication bias, and the lack of access to unpublished or incomplete data may have influenced the aggregate results (58).

### 4.1 Limitations of the meta-analysis

The present review also has some limitations. First, the present study was restricted to eligibility criteria, in which merely “Number of studies” were included in the analysis. Some unpublished and missing data from studies also influence aggregate results. Furthermore, some of the studies were completed by the same researchers, which may lead to publication bias. In addition, the double-blind period of these included studies ranged from 3 to 6 months, and the difference might result in heterogeneity. To reduce heterogeneity, only studies with 12-week follow-up were included in the final quantitative synthesis. While this approach improved comparability across trials, it also excluded a significant number of potentially relevant studies and may have limited the scope of the analysis, particularly with regard to long-term efficacy and safety outcomes.

Additionally, pharmacological treatments were grouped into broad therapeutic classes (e.g., antidepressants, anti-seizure medications), despite marked differences in their mechanisms of action and clinical profiles. For example, amitriptyline and venlafaxine, although both classified as antidepressants, have distinct pharmacodynamic properties; similarly, topiramate and valproate differ substantially in their molecular targets and tolerability. This classification may oversimplify treatment effects and obscure clinically meaningful differences between individual agents. Greater granularity, as reflected in the compound-specific data provided in Supplementary material, is likely to be more informative for guiding clinical decision making. Also, the analysis excluded older, non-specific pharmacological therapies commonly used in migraine prevention—such as other beta-blockers and certain calcium channel blockers—owing to the lack of recent or high-quality RCTs meeting inclusion criteria. Some of them are treatments that remain widely prescribed in routine practice in some countries, and their omission from the current synthesis may limit the generalizability of the findings.

In addition, non-pharmacological and nutraceutical interventions were grouped into heterogeneous categories, despite having distinct therapeutic mechanisms and varying levels of supporting evidence. This broad classification complicates interpretation and precludes firm conclusions about the relative efficacy of individual non-drug strategies.

Moreover, this review included only studies published in English or Spanish, which may have introduced language bias and limited the inclusion of potentially relevant trials from other regions. Otherwise, Meta-analyses were conducted using SPSS due to software availability at the institution. While SPSS is appropriate for basic fixed- and random-effects models, it does not offer the advanced options or flexibility of specialized platforms such as RevMan or R-based packages like meta or metafor. This may limit some statistical nuance in modeling or subgroup analysis.

Finally, due to the exclusion of patients older than 65 years, pregnant individuals, and those with significant cardiovascular or cerebrovascular comorbidities, the generalizability of the results is limited. These populations, which are frequently encountered in clinical practice, remain underrepresented in current trials. Moreover, future studies should prioritize the investigation of subgroup-specific responses to preventive treatments, including stratification by migraine frequency (e.g., high-frequency episodic vs. chronic migraine), sex, and age group. Such analyses are essential to advancing a more tailored and equitable approach to migraine management.

Furthermore, studies with longer follow-ups and larger sample sizes should be performed to identify the confirmative safety profile of gepants and monoclonal antibodies and determine the duration of its therapeutic effects.

Taken together, these limitations highlight the need for further high-quality, head-to-head trials of both pharmacological (e.g., gepants vs. monoclonal antibodies) and non-pharmacological treatments, with mechanistic specificity, standardized outcomes, and longer follow-up durations, and evaluations of cost-effects of the treatment to better inform personalized approaches to migraine prevention.

### 4.2 Bullet points

- Preventive therapy for episodic migraine should be individualized.
- Combined strategies (pharmacological + non-pharmacological) are recommended.
- Decision-making should consider comorbidities, adverse effect profiles, and patient preferences.

## 5 Conclusion

This systematic meta-analysis highlights the efficacy and safety of preventive treatments for episodic migraine, with a focus on their applicability in Mexico. While active treatments showed an overall favorable effect (mean difference: –1.25; 95% CI: −1.47 to −1.04; *p* = 0.001). with a variability in response. Anti-CGRP monoclonal antibodies and gepants were associated with clinically meaningful reductions in MDD and acceptable safety, offering advantages over conventional therapies. Traditional agents, including beta-blockers and antiepileptics, showed more modest efficacy and tolerability, while pharmacological combinations, though effective, were limited by side effects. Non-pharmacological strategies showed promise but lacked consistent evidence.

The limited availability of newer therapies in Mexico highlights the need for national strategies to expand formulary access and align with international treatment standards. Methodological limitations—including the exclusion of older adults and pregnant individuals, short follow-up periods, and variability in drugs. Future research should prioritize inclusive, long-term, and head-to-head trials to better inform personalized, evidence-based migraine prevention.

## Data Availability

The original contributions presented in the study are included in the article/Supplementary material, further inquiries can be directed to the corresponding author.
